# Twenty‐Four Month Outcomes From a Real‐World Telehealth Obesity Treatment Clinic Using Obesity Medications

**DOI:** 10.1002/oby.70156

**Published:** 2026-03-01

**Authors:** Kathleen R. Ruddiman, Young‐Rock Hong, Jamy Ard, Rebecca Jones, Adam Medcalf, Gary D. Foster, Jamil Alkhaddo, Michelle Cardel

**Affiliations:** ^1^ Department of Surgery, Bariatric and Weight Management Section Wake Forest University School of Medicine Winston‐Salem North Carolina USA; ^2^ Department of Family and Preventive Medicine Emory University School of Medicine Atlanta Georgia USA; ^3^ WW International, Inc. New York New York USA; ^4^ Center for Weight and Eating Disorders, Perelman School of Medicine University of Pennsylvania Philadelphia Pennsylvania USA; ^5^ Department of Health Outcomes and Biomedical Informatics University of Florida College of Medicine Gainesville Florida USA; ^6^ Center for Integrative Cardiovascular and Metabolic Disease University of Florida Gainesville Florida USA

**Keywords:** AOM, obesity medication, real‐world outcomes, telemedicine clinic

## Abstract

**Objective:**

Antiobesity medications (AOMs) are indicated for long‐term use; however, evidence of their real‐world long‐term efficacy is limited. This study describes clinical outcomes from 18‐ and 24‐month use of AOMs in a telemedicine weight management clinic.

**Methods:**

This retrospective observational study analyzed data from adult participants who utilized AOMs at a telemedicine weight management clinic between January 2022 and December 2024. Weight change analyses were conducted for those maintaining AOM use for ≥ 18 months (*n* = 11,675) and ≥ 24 months (*n* = 4317). Weight outcomes were assessed across all AOMs and stratified based on classification of AOMs utilized.

**Results:**

Across all AOMs, average total body weight reduction of −18.53% (95% CI: −18.74%, −18.32%) was observed at 18 months and −20.27% (95% CI: −20.63%, −19.92%) seen at 24 months. For those taking long‐acting AOMs, outcomes trended toward greater weight loss in comparison with participants using oral AOMs.

**Conclusions:**

This real‐world analysis found 18‐ and 24‐month weight losses comparable to those demonstrated in clinical trials, supporting the long‐term efficacy of AOMs provided by a telemedicine weight management clinic. Continued weight loss was demonstrated between 18 and 24 months, suggesting sustained engagement with AOMs and a telemedicine weight management clinic support continued weight loss in the long term.

## Study Importance

- What is already known?
  - Use of antiobesity medications (AOMs) has increased dramatically over the last several years, along with options for virtual‐based obesity treatment.
  - However, there is little data on outcomes from long‐term utilization of AOMs through these real‐world programs.
- What does this study add?
  - Real‐world use of AOMs in a telehealth‐based clinic resulted in sustained, significant average weight loss of 18.53% at 18 months and 20.27% at 24 months.
- How might these results change the direction of research or the focus of clinical practice?
  - These results suggest that telemedicine is an effective platform for providing obesity treatment on a large‐scale basis, with outcomes similar to phase 3 trials and other in‐person treatment settings.

## 1 Introduction

Based on US Centers for Disease Control and Prevention (CDC) data from 2021 to 2023, the prevalence of obesity (defined by BMI ≥ 30 kg/m^2^) among adults in the US was 40.3%, with 9.4% representing severe obesity (BMI ≥ 40 kg/m^2^) [1]. Despite being recognized as a chronic disease by the National Institutes of Health since 1998 [2], only a small percentage of individuals who qualify for antiobesity medications (AOMs) have been prescribed these treatments. Prescribing data from over 2.2 million people revealed that only 1.3% of patients eligible for AOM therapy received a prescription between 2009 and 2015 [3]. However, prescriptions for AOMs have been increasing in recent years, particularly for the glucagon‐like peptide‐1 receptor agonists (GLP‐1 RAs) and glucagon‐like peptide‐1/gastric inhibitory peptide receptor agonists (GLP‐1/GIP RAs). Prescription fills for semaglutide for obesity treatment at commercial pharmacies increased by 1361% between July 2021 and December 2023 [4]. By February 2024, prescriptions for semaglutide and tirzepatide for obesity treatment had respectively reached 0.42 and 0.25 million per month, in another population‐level analysis [5].

Barriers to treating obesity include under‐referral to weight management centers, patient and health care provider perceptions about initiating obesity treatment, insurance coverage of AOMs, and lack of access to health care providers trained in obesity treatment and multidisciplinary treatment teams [6, 7]. Of these barriers, access to obesity care has improved with increasing telemedicine utilization during the COVID‐19 pandemic. Between 2019 and 2021, use of telemedicine rose from 15.4% to 85.9% among US office‐based physicians [8]. Despite growing evidence of telemedicine use for obesity treatment [9, 10, 11, 12, 13] and recent increases in AOM prescribing, there is little current data on long‐term outcomes resulting from the combination of these tools. This study describes clinical outcomes from a widely available telehealth obesity treatment program using AOMs in a large cohort of patients at 18 and 24 months, including weight loss outcomes, medication usage patterns, and side effect patterns during the course of treatment.

## 2 Methods

### 2.1 Study Design, Inclusion and Exclusion Criteria

This was a retrospective review of data collected from a widely available telehealth obesity medicine provider (WeightWatchers [WW] Clinic) that included adult patients who initiated treatment between January 2022 and December 2024 and met Food and Drug Administration (FDA) guidelines for AOM use: BMI ≥ 30 kg/m^2^ or BMI ≥ 27 kg/m^2^ with a comorbid condition, including hypertension, prediabetes, hyperlipidemia, or obstructive sleep apnea. Exclusion criteria included age < 18 years, ineligibility for AOM treatment based on FDA criteria, type 1 diabetes, pregnancy, known eating disorder diagnoses (such as anorexia or bulimia nervosa), or residence outside of the US.

The current study was a new analysis of all WW Clinic members who had completed 18 and 24 months of treatment in December 2024, rather than a follow‐up analysis of the previous cohort of participants reaching 12 months of treatment, whose outcomes were described previously [14]. Because deidentified health records were used for data analysis, this study was considered exempt by the Institutional Review Board at the University of Florida (NH00021333). This study aimed to quantify the weight loss outcomes and side effects associated with real‐world long‐term use of AOMs in the setting of a telehealth obesity medicine provider.

### 2.2 Treatment Program

In order to enroll with WW Clinic, prospective patients completed an online intake survey to determine if they met FDA guidelines for AOM use. Patients provided photographs of weight measurements on their at‐home scales for weight verification. Following the online screening, qualified patients met virtually with a board‐certified clinician with training in obesity pathophysiology and pharmacology, obesity bias and stigma, and case review and policy updates, as well as electronic medical record training. Clinicians assessed which patients would be medically appropriate to receive AOMs, which were then prescribed after a shared decision‐making discussion based on relevant medical history, patient goals, and insurance coverage. AOMs offered by the WW Clinic were categorized as long‐acting, short‐acting, or oral medications. Long‐acting AOMs included semaglutide (injectable), dulaglutide, and tirzepatide; short‐acting AOMs included liraglutide; oral AOMs included bupropion/naltrexone, metformin, and oral semaglutide at a dose of 7 or 14 mg. Controlled AOMs including phentermine/topiramate were not available through WW Clinic. Given their weight loss benefits, non‐FDA‐approved medications (metformin, dulaglutide, and oral semaglutide) were included for either off‐label use or were prescribed in the setting of type 2 diabetes.

Following clinic enrollment, patients' progress was regularly evaluated by their clinician including monitoring weight change, mental health, side effects, and other relevant information. Patients had access to registered dietitians and fitness specialists to develop personalized nutrition and exercise plans, as well as access to the WW behavioral programs. The WW behavioral programs include the trademark Points program, the GLP‐1 Companion tailored program for those on GLP‐1 RAs, and the Diabetes program. The behavioral programs support self‐monitoring of weight, dietary intake, and physical activity. The WW app features instant messaging with a patient's care team, a tool for prescription refill requests, unlimited coach‐led WW Workshops, and support via Connect (members‐only community). The WW Clinic program included all clinical care, fitness consults, dietitian visits (insurance dependent or cash pay options), prior authorization of AOMs, assistance with location of medications when supply was limited, and the costs of generic and non‐schedule IV AOMs (bupropion/naltrexone and metformin). If medication costs (outside of bupropion/naltrexone and/or metformin) were not covered by insurance, patients paid out of pocket.

### 2.3 Data Collection and Outcome Measures

Data used in the retrospective analysis were routinely collected at clinician visits and from self‐reported data in the app. This included weight, AOM‐related side effects, and first and last AOMs used. Baseline height and weight were collected by patient self‐report at the initial intake survey and photo verification of scale weight was used at baseline. Follow‐up weights and AOM side effects were reported through the WW app prior to prescription refill. First AOM type was defined as the first prescription provided to the participant. This was initially captured using prescription history and confirmed with refill history; those with no refill history were removed from the analytic cohort. Last AOM types were also collected based on active prescriptions in the WW app within a 15‐day window of the 18‐ or 24‐month mark. Adverse effects were reported under the symptom categories abdominal pain, appetite loss, constipation, diarrhea, hair loss, injection site reactions, nausea, and other. Patients who had continued program participation and AOM use at 18 and 24 months were included for analysis; those who had both 18‐ and 24‐month data at the end of the follow‐up period were included for analysis in both groups. Primary outcome measures included absolute weight change (kilograms) and percentage change from baseline at 18 and 24 months after treatment initiation, as well as monthly rates of AOM side effects.

### 2.4 Statistical Analysis

Descriptive statistics were generated to characterize baseline demographics and clinical characteristics of the study sample. Weight change analyses employed paired comparison methods, as weight measurements at follow‐up time points (18 and 24 months) were compared to baseline values within the same individuals. Mean weight changes (in kilograms) and percentage changes from baseline were calculated as within‐subject differences, with 95% confidence intervals (CI) computed using the t‐distribution based on the standard error of the mean difference. We evaluated treatment response using predefined categorical thresholds of 5%, 10%, 15%, 20%, and 25% weight reduction from the baseline. We conducted stratified analyses to examine differential weight outcomes based on medication classifications (long‐acting, short‐acting, and oral AOMs) and longitudinal medication utilization patterns (i.e., transitions between initial and final AOM categories). We employed complete case analysis for the study endpoints. All statistical analyses were conducted using SAS version 9.4 (SAS Institute Inc., Cary, North Carolina) from December 2024 to January 2025.

## 3 Results

**FIGURE 1:**
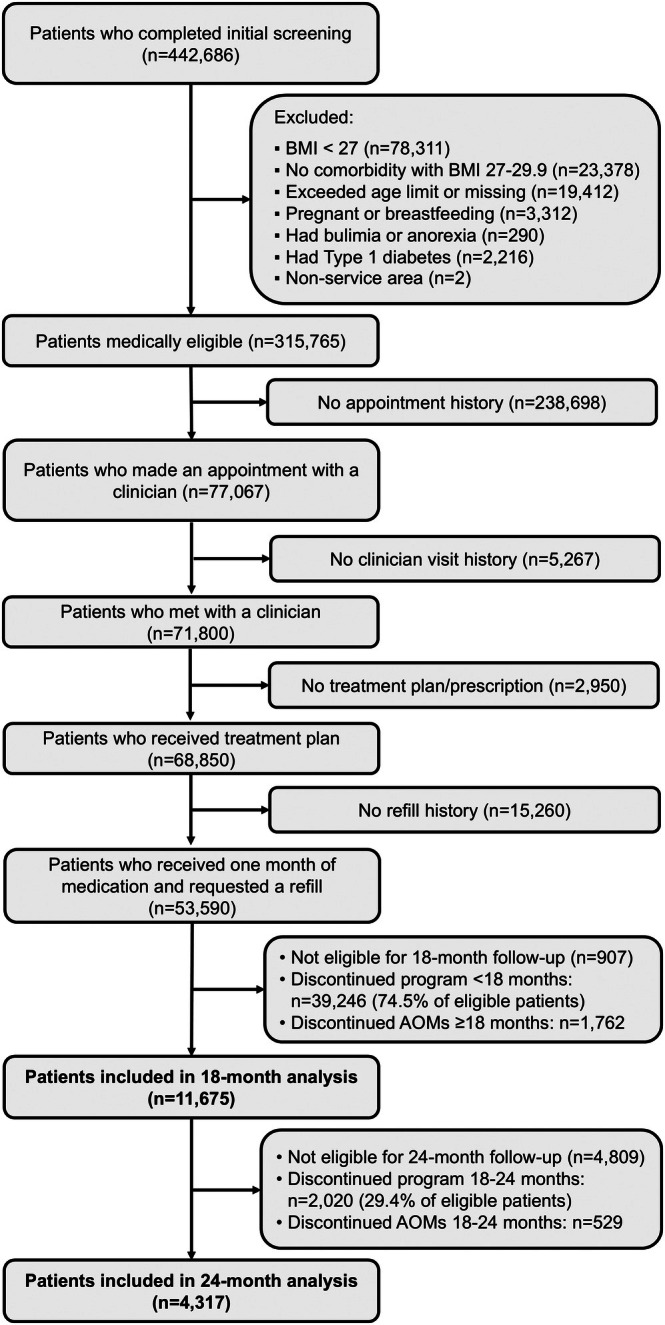
WeightWatchers clinic enrollment flowchart. Participants whose membership duration allowed for both 18‐ and 24‐month data collection were included in both analyses. For retention calculations, total participants eligible for analysis at 18 months: *n* = 52,683; at 24 months: *n* = 29,868.

The analytic sample selection process is shown in Figure 1. Initially, 442,686 prospective patients completed the program eligibility survey. Of 315,765 patients who met initial inclusion criteria, 53,590 met with a clinician and received a treatment plan followed by at least one prescription refill request after 1 month of AOM treatment. From the group that initiated treatment, those who completed 18 months (*n* = 11,675) and 24 months (*n* = 4317) of membership with AOM use (as indicated by continued AOM use at those time cutoffs) were compared to participants who could have been enrolled for at least 18 months (*n* = 52,683) and 24 months (*n* = 29,868), resulting in retention rates of 22.16% at 18 months and 14.45% at 24 months.

Baseline participant characteristics are shown in Table 1. Of those who completed 18 and 24 months of WW Clinic treatment, the average age was 43 years and the groups were composed of 87.91% and 90.55% female participants, respectively. Participants' average starting weight was 101.19 kg (BMI 36.4 kg/m^2^) in the 18‐month group and 102.21 kg (BMI 37.0 kg/m^2^) within the 24‐month group. Over 99% of those who enrolled reported previously following a diet plan for weight loss and using an average of 2.9 different diet types prior to enrolling in the telemedicine program. A comparison of baseline demographics between those who completed 18 or 24 months of AOM use and participants who discontinued AOMs prior to those cut points can be found in Table S1. Participants who discontinued prior to 18 and 24 months were younger by about 2 years in both groups (*p* < 0.001). There was a higher prevalence of polycystic ovarian syndrome in the groups who discontinued prior to 18 months (*p* = 0.001) and 24 months (*p* = 0.018).

**TABLE 1:** Study sample baseline characteristics.

	Those on AOM at least 18 months	Those on AOM at least 24 months
Total no.	*n* = 11,675	*n* = 4317
Age, years, mean (SD)	43.16 (9.51)	43.44 (9.11)
Sex at birth, %		
Female	87.91%	90.55%
Male	12.09%	9.45%
Weight, kg, mean (SD)	101.19 (20.13)	102.21 (20.81)
BMI, mean (SD)	36.47 (6.29)	37.01 (6.55)
No. of previous diets tried, mean (SD)	2.91 (1.18)	2.95 (1.18)
Type of weight loss tried in the past, %		
Dieting	99.12%	99.14%
Exercise plan	91.63%	91.98%
Coaching	53.46%	55.19%
Medications	39.96%	44.30%
Bariatric surgery	7.43%	7.46%
Other	8.90%	9.22%
Previous diagnosis, %		
Type 2 diabetes	3.19%	3.76%
Obstructive sleep apnea	10.59%	11.03%
Low HDL	5.48%	5.31%
High triglycerides	16.07%	15.44%
Prediabetes	16.78%	16.16%
Hypertension	23.18%	22.35%
Heart disease	0.86%	0.88%
Polycystic ovarian syndrome^a^	15.15%	15.16%
Osteoarthritis	3.57%	3.30%
Nonalcoholic fatty liver disease	4.09%	4.31%
Urinary incontinence	1.26%	2.32%
Gastroesophageal reflux disease	5.74%	10.34%
None of these	44.30%	42.85%

Abbreviations: AOM, antiobesity medication; HDL, high‐density lipoprotein; SD, standard deviation.

^a^ Only females included.

Mean weight change from baseline at 18 months was −19.19 kg (95% CI: −19.44, −18.95) or −18.53% (95% CI: −18.74%, −18.32%). At 24 months, mean weight change from baseline was −21.29 kg (95% CI: −21.72, −20.86) or −20.27% (95% CI: −20.63%, −19.92%) (Table 2). Rates of > 5%, > 10%, > 15%, > 20%, and > 25% total weight loss among participants at 18 and 24 months can be found in Table 2. A majority of participants, totaling 91.0% (*n* = 10,626) of the group at 18 months and 91.6% (*n* = 3954) at 24 months, experienced weight loss of 5% during program engagement. At 18 months, 64.5% (*n* = 7532) of participants had weight loss of 15%, and 26.3% (*n* = 3072) had weight loss of 25%. At 24 months, 67.7% (*n* = 2924) experienced weight loss of 15% and 31.4% (*n* = 1354) had weight loss of 25%.

**TABLE 2:** Weight change outcomes at 18 and 24 months.

	Those on AOM at least 18 months	Those on AOM at least 24 months
Total no.	*n* = 11,675	*n* = 4317
Baseline weight		
Mean weight, kg, mean (SD)	101.19 (20.13)	102.21 (20.81)
Outcomes		
Mean weight, kg (SD)	81.99 (18.48)	80.92 (18.56)
Change from baseline, kg (95% CI)	−19.19 (−19.44 to −18.95)	−21.29 (−21.72 to −20.86)
Change from baseline, % (95% CI)	−18.53% (−18.74 to −18.32)	−20.27% (−20.63 to −19.92)
Achieved > 5% target, no. (%)	10,626 (91.0)	3954 (91.6)
Achieved > 10% target, no. (%)	9436 (80.8)	3535 (81.9)
Achieved > 15% target, no. (%)	7532 (64.5)	2924 (67.7)
Achieved > 20% target, no. (%)	5243 (44.9)	2197 (50.9)
Achieved > 25% target, no. (%)	3072 (26.3)	1354 (31.4)

Long‐acting AOMs were the most frequently prescribed medication type at both 18 and 24 months (Table 3A,B). In the 18‐month cohort and 24‐month cohort, respectively, 9836 participants (84.2%) and 3503 (81.1%) were using long‐acting AOMs at both the start and end of the study. Among 18‐month and 24‐month participants who initially received oral AOMs (*n* = 1203 at 18 months, *n* = 646 at 24 months), 84.7% (*n* = 1021) and 90.7% (*n* = 586) had transitioned to long‐acting AOMs by the end of the study. Among patients with continuous AOM use, 44% remained on tirzepatide, 16.7% remained on semaglutide, 13.3% switched from semaglutide to tirzepatide, and 9.8% switched from tirzepatide to semaglutide. At the end of the study, between 93.5% and 95.5% of all participants were using long‐acting AOMs. Only 1.4%–1.6% of participants used oral AOMs at both the start and end of the study. The least frequently prescribed AOM class was short‐acting AOMs (i.e., liraglutide); all patients who had started liraglutide initially transitioned to a different AOM type by the end of the study.

**TABLE 3:** Weight changes at 18 and 24 months by first and last AOM combination.

Initial medication	Last medication								
	Long‐acting			Short‐acting			Oral		
	No.	Weight change, kg (95% CI)	Weight change, % (95% CI)	No.	Weight change, kg (95% CI)	Weight change, % (95% CI)	No.	Weight change, kg (95% CI)	Weight change, % (95% CI)
**(A) 18 months**									
Long‐acting	9836	−19.4 (−19.66 to −19.13)	−18.75% (−18.97 to −18.52)	—	—	—	571	−18.30 (−19.49 to −17.12)	−17.32% (−18.31 to −16.33)
Short‐acting	61	−20.76 (−23.89 to −17.63)	−19.98% (−22.71 to −17.26)	—	—	—	4	−11.61 (−34.25 to 11.03)	−11.73% (−37.17 to 13.72)
Oral	1021	−18.62 (−19.44 to −17.79)	−18.07% (−18.76 to 17.38)	—	—	—	182	−13.89 (−16.13 to −11.66)	−12.95% (−15.00 to −10.89)
**(B) 24 months**									
Long‐acting	3503	−21.5 (−21.97 to −21.03)	−20.48% (−21.97 to −21.03)	—	—	—	136	−21.8 (−24.61 to −18.99)	−19.84% (−21.90 to −17.77)
Short‐acting	32	−23.62 (−28.55 to −18.69)	−21.94% (−25.70 to 18.17)	—	—	—	—	—	—
Oral	586	−20.43 (−21.69 to −19.17)	−19.55% (−20.53 to −18.56)	—	—	—	60	−15.14 (−18.19 to −12.09)	−15.35% (−17.95 to −12.76)

*Note*: Long‐acting AOM includes GLP‐1 receptor agonists that are administered weekly via subcutaneous injection. Included medications are semaglutide (Wegovy/Ozempic), dulaglutide (Trulicity), and tirzepatide (Mounjaro/Zepbound). Short‐acting AOM includes liraglutide (Saxenda/Victoza) requiring daily subcutaneous injection. Oral AOM includes oral semaglutide (Rybelsus), naltrexone/bupropion (Contrave), and metformin.

When grouped by first and last AOMs used, those who ended the study period using long‐acting AOMs had a similar percentage of weight loss, regardless of their starting medication. For those ending treatment on long‐acting AOMs, the percentage weight loss ranged between 18.07% and 19.98% at 18 months and between 19.55% and 21.94% at 24 months. For those starting and ending on long‐acting medications, mean weight change was −19.4 kg (95% CI: −19.66, −19.13) at 18 months (−18.75%; 95% CI: −18.97%, −18.52%) and −21.5 kg (95% CI: −21.97, −21.03) at 24 months (−20.48%; 95% CI: −21.97%, −21.03%). In the 18‐month cohort, four patients who started the program using the short‐acting AOM liraglutide ended the program using an oral agent. In the 24‐month cohort, all participants who started on a short‐acting agent had transitioned to a long‐acting AOM (*n* = 32). The mean number of AOM refills for active WW Clinic participants was 16.8 (SD 6.5) at 18 months of participation and 19.8 (SD 6.9) at 24 months.

**FIGURE 2:**
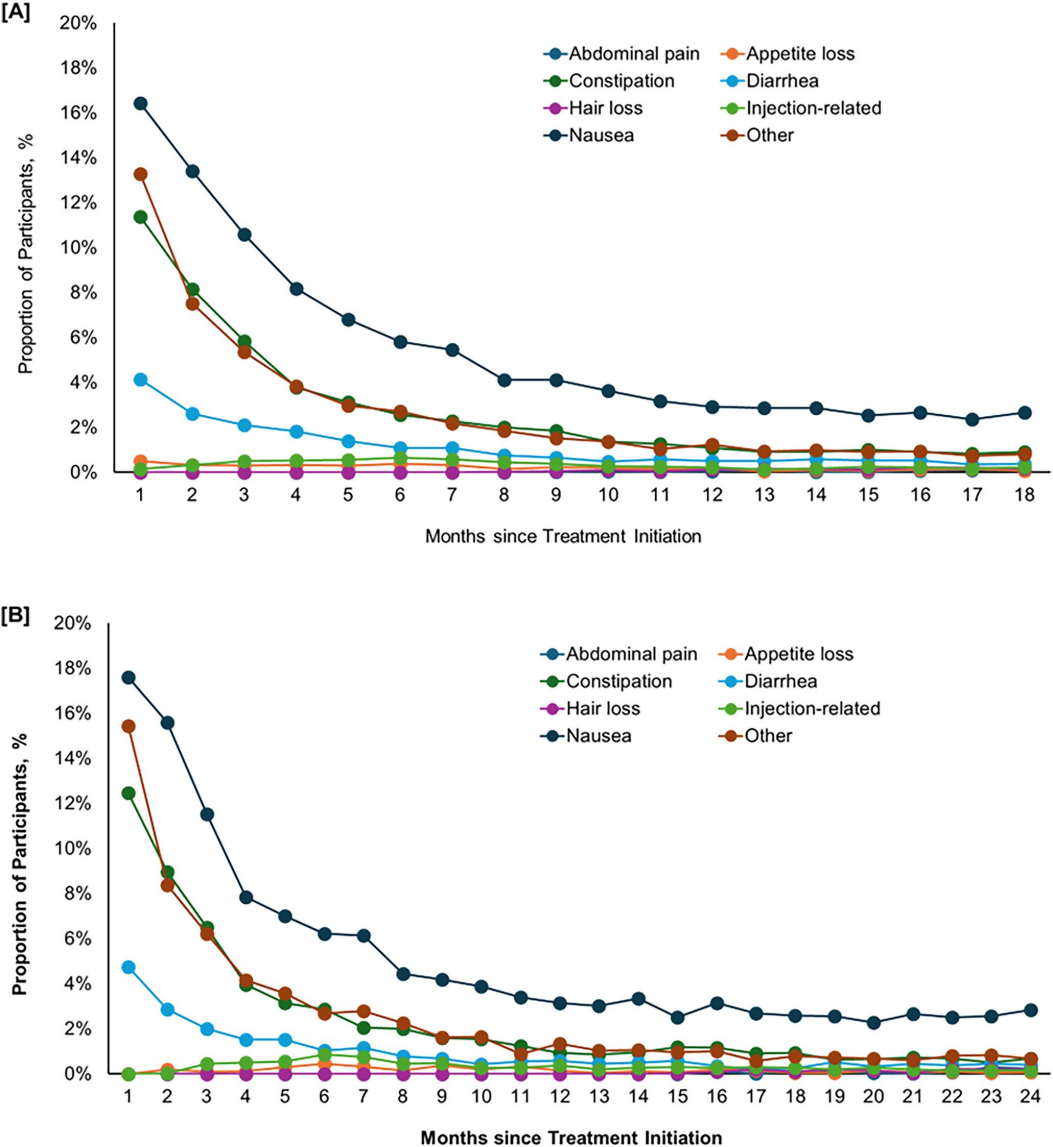
Monthly prevalence of side effects among participants with (A) 18‐month follow‐up and (B) 24‐month follow‐up. Other side effects included: heartburn/reflux, fatigue, headache, dizziness, muscle aches, and sleep disturbances. [Color figure can be viewed at wileyonlinelibrary.com]

Adverse effects at 18 and 24 months are depicted in Figure 2A,B. Nausea was the most frequently reported side effect in both the 18‐ and 24‐month cohorts, followed by “other” symptoms, constipation, and diarrhea, respectively. The “other” side effect category captured symptoms that did not fall into the prespecified side effect categories and most commonly included heartburn or reflux, fatigue, headache, dizziness, muscle aches, and sleep disturbances. More details regarding monthly rates of adverse effects reported can be found in Tables S2 and S3.

## 4 Discussion

This study provides valuable long‐term data on real‐world use of AOMs delivered through a telehealth obesity treatment program. On average, participants lost 18.53% of total body weight at 18 months and 20.27% at 24 months. About two‐thirds of participants in both groups had greater than 15% weight loss, and over a quarter of participants in both groups experienced over 25% weight loss. A variety of AOMs were used, with the most frequently used being the long‐acting AOMs semaglutide and tirzepatide. The most commonly reported gastrointestinal side effects were nausea, constipation, and diarrhea, and all of these decreased in frequency over time. Compared with previously reported WW Clinic weight loss outcomes of 19.4% at 12 months [14], WW Clinic participants demonstrated continued weight loss at 24 months.

The observed weight loss outcomes among WW Clinic participants were higher than weight loss in phase 3 trials of semaglutide 2.4 mg [15] and comparable to phase 3 outcomes from tirzepatide 15 mg [16] for obesity treatment. WW Clinic participants experienced greater weight loss compared to clinical trials of other injectable GLP‐1 RA medications including daily liraglutide 3.0 mg for obesity [17] and dulaglutide 4.5 mg weekly used in patients with type 2 diabetes and overweight or obesity [18], as well as oral AOMs including naltrexone with bupropion [19], oral semaglutide used in patients with type 2 diabetes [20], and metformin [21]. One likely explanation for these findings is the higher proportion of long‐acting AOM use (semaglutide and tirzepatide) in the WW Clinic, with 44% remaining on tirzepatide and 16.7% on semaglutide for the duration of follow‐up, and another 23.1% who switched between long‐acting AOM agents during the follow‐up period. Overall, weight loss outcomes, suspected to be largely reflective of long‐acting AOM use, suggest that semaglutide and tirzepatide had equal or better real‐world efficacy compared to phase 3 trials when used over 24 months within a telehealth obesity treatment program.

Recent data have demonstrated the efficacy of other telehealth obesity treatment programs using behavioral interventions alone [22, 23, 24, 25, 26, 27] and lifestyle interventions combined with AOMs [9, 10, 28]. In comparison with WW Clinic outcomes, other telehealth obesity treatment programs using AOMs have produced variable weight loss outcomes. One retrospective study reported weight loss of 16.21% in participants who completed between 12 and 18 months of treatment combining semaglutide or tirzepatide with telemedicine visits and self‐monitoring using a mobile app [10]. Weight loss of 14.48% was observed among those who used “first‐generation AOMs” (any AOM aside from semaglutide or tirzepatide) [10]. Other virtual weight loss interventions using AOMs observed average participant weight loss of 5.8% at 6 months [28] and 8% at 12 months [9]. Trends of higher weight loss outcomes are seen across studies with higher long‐acting AOM use compared with higher oral AOM use [9, 10, 28].

Outcomes from real‐world AOM use delivered through in‐person follow‐up also varied compared to the WW Clinic findings. Paddu et al. recently reported on the effects of 12‐month GLP‐1 RA use followed by transition to generic oral AOMs for maintenance within a multidisciplinary weight loss program. In a prospective analysis, patients experienced weight loss of 25% at 18 months and 16% at 24 months [29]. Within the WW Clinic cohort, those who followed a similar treatment trajectory by starting treatment on long‐acting AOMs and ending on oral medication had weight loss of 17% and 19% at 18 and 24 months, reflecting at least comparable 24‐month outcomes. Another in‐person program using AOMs reported lower weight loss outcomes, with weight loss of 3.4% at 12 months and 2.7% at 24 months, though this analysis was conducted prior to FDA approval of GLP‐1 RA or GLP‐1/GIP RA medications for obesity treatment [30]. Finally, meta‐analysis of multiple obesity treatment centers using AOMs between 2016 and 2020 reported weight loss of 10.5% at 24 months [31]. With significant variability between real‐world outcomes among both clinical platforms, more data are needed to elucidate outcomes from in‐person AOM use and telemedicine‐based AOM use.

There were many strengths and limitations of this analysis. This study was strengthened by a large sample size; the WW Clinic had a group of over 11,000 participants for analysis. By offering a wide variety of AOMs, the study was more reflective of other real‐world obesity treatment clinics, increasing generalizability. The extended follow‐up period was another strength; few other studies reported on a telemedicine‐based intervention followed for a 24‐month duration [10]. One limitation was that the WW Clinic population was mainly female, though this is notably similar to other populations using AOMs for weight loss in real‐world studies [28, 32, 33, 34] and in several phase 3 trials [15, 16, 17]. WW Clinic participants did not report ethnicity information, which limits generalizability. Use of self‐reported weights represents an area of potential bias due to previously observed patterns of weight underreporting, particularly among women [35]. Self‐reported weight has good correlation with image‐captured weights [36]. While self‐report with scale images is a validated and practical approach in large‐scale behavioral studies [36], selective reporting and small systematic differences may arise; however, in the context of the profound weight changes observed in this sample, we expect any such biases to be minimal and unlikely to meaningfully alter the interpretation of study findings at the group level. Another area of bias stems from participant self‐selection to participate with the WW Clinic; there may be significant differences between WW Clinic participants who terminated participation compared to those who maintained active AOM use and membership for 18–24 months, which may bias the outcomes away from the null. As this was an observational study, we were limited in controlling for this bias, but future studies of weight management clinic platforms may consider comparison between clinic participants and a control population receiving AOMs from various other platforms to adjust for participant variability.

The retrospective, observational nature of the study presents several further limitations to the scope of data collected from clinic participants. The WW Clinic did not track outcomes from participants who discontinued WW Clinic membership, which represents a large proportion of initial program enrollees. It is important to note that WW Clinic membership discontinuation does not necessarily reflect AOM discontinuation; those who terminated WW Clinic membership may have discontinued AOMs, taken an interim break from AOM use, or continued AOMs prescribed by other providers. Collecting data from this group in future studies would help illustrate the natural history and outcomes of AOM use in the real world and may also help clarify reasons for program discontinuation in order to improve long‐term program utilization. The study also did not have a definite mechanism to denote use of multiple AOMs at once during the study period; doing so would provide a deeper insight into real‐world medication usage patterns and the differential effectiveness of combination AOM approaches. Additionally, WW Clinic data collection limited the ability to correlate side effect timing with AOM type used at the time each side effect was reported, a detail which may also help improve our understanding of potential barriers to long‐term AOM use. Finally, there was not a mechanism in place to ensure that participants were self‐monitoring or adhering to lifestyle change recommendations, as not all participants had free access to use the app. This limitation may result in weight loss outcomes being more strongly reflective of AOM use, since participant adherence to lifestyle modifications could not be verified. Further prospective, longitudinal investigation of real‐world AOM use is needed to clarify some of these questions that this dataset could not elucidate.

A last limitation of the study was low participant retention rates. As already noted, participant attrition is not equivalent to AOM discontinuation; however, significant rates of AOM discontinuation have been observed in other examples of real‐world AOM use [34, 37, 38] and may carry significant ramifications for those who enroll with weight loss programs intending to initiate long‐term AOM treatment. In other real‐world settings, retrospective pharmacy claim‐based studies reported similar discontinuation rates of GLP‐1 RA treatment for overweight or obesity, from 50% to 68% at 12 months [34, 37, 38] up to 84% at 24 months [34] in patients without diabetes. This suggests that poor levels of treatment adherence are not uncommon; however, more data are needed to identify which aspects of care may help improve retention in weight loss programs such as the WW Clinic. Despite low retention rates, refill data from active WW Clinic participants suggest high levels of adherence for those who completed 18 and 24 months of active clinic membership, with the average number of refills nearing the total months of participation (16.8 [SD 6.5] at 18 months and 19.8 [SD 6.9] at 24 months). However, the program did not distinguish between average refill requests in patients receiving multiple AOMs, which is a confounding factor for interpreting this data and may falsely elevate presumed adherence if representing refills of multiple medications; however, this does explain why standard deviations from the mean refill number may exceed total months of participation. Collecting data on the number of monthly refills of each AOM type used would help clarify the question of AOM adherence during active program membership.

This study introduces several questions for future investigation. More data are needed to confirm the efficacy of long‐term AOM use in real‐world settings and to explore concepts such as cost‐effectiveness, side effects, tolerability, causes for medication discontinuation, and other barriers to long‐term AOM utilization. Prospective studies are also needed to observe outcomes in those who discontinue or have interrupted AOM use, as is commonly seen in real‐world scenarios. Finally, additional studies with more diverse populations that mirror the general population eligible for AOM treatment are needed.

In conclusion, this study reflects the long‐term efficacy of a real‐world telehealth clinic for treatment of obesity using AOMs at 18 and 24 months. Weight loss efficacy is comparable to those observed in phase 3 trials of semaglutide and tirzepatide, with fewer adverse effects reported as well. The telehealth platform appears to be an effective and durable mode of delivering medical care and pharmacotherapy with clear weight loss benefits for those who continue AOMs over long‐term periods of use.

## Funding

This work was supported by WW International, Inc.

## Conflicts of Interest

Y.R.H. reports receiving consulting fees from WW International, Inc. for consultation on study design and statistical analysis. J. Ard reports receiving grants or contracts from Nestle Healthcare Nutrition, Eli Lilly, Boehringer Ingelheim, Epitomee, Inc., UnitedHealth Group R&D, KVKTech, WW International, Inc., and Regeneron; receiving consulting fees from Nestle Healthcare Nutrition, Eli Lilly, Optum Labs R&D, Novo Nordisk, Intuitive, Regeneron, Brightseed, WW International, Inc., Amgen, and Boehringer Ingelheim; holding leadership or fiduciary roles for The Obesity Society (President) and the American Society for Nutrition Foundation (Executive Board Member); and receipt of equipment, materials, drugs, medical writing, gifts, or other services from KVK Tech, WW International, Inc., and Nestle Healthcare Nutrition. AM reports employment by WW International, Inc., and holding shares/stock options in WW International, Inc. GDF reports employment by WW International, Inc., from July 2013 to December 31, 2024, and support for attending meetings and/or travel during employment by WW International, Inc.; receiving consulting fees from WW International, Inc., starting January 1, 2025; and holding stock or stock options of WW International, Inc. J. Alkhaddo reports receiving consulting fees from North County Endocrinology Associates for general endocrinology services; receiving payment for expert testimony from Maximus Federal services as an independent medical reviewer; and holding leadership or fiduciary roles for Tricity Hospital (Medical Director) providing diabetes services. M.C. reports employment by WW International, Inc.; holding leadership or fiduciary roles in WW International, Inc.; and holding stock or stock options in WW International, Inc. The other authors declared no conflicts of interest.

## Supporting information


**Table S1:** Demographic Comparison of Participants Completing 18 and 24 months of WW Clinic Participation versus Participants who Discontinued Antiobesity Medication (AOM).
**Table S2:** Monthly Reported Adverse Effects and Prevalence in 18‐Month Cohort.
**Table S3:** Monthly Reported Adverse Effects and Prevalence in 24‐Month Cohort.

## Data Availability

The data that support the findings of this study are available from the corresponding author upon reasonable request.
