# Supplementary material for: Twenty‐Four Month Outcomes From a Real‐World Telehealth Obesity Treatment Clinic Using Obesity Medications
Source: Obesity (Silver Spring). 2026 Mar 1;34(Suppl 1):134–43. doi: 10.1002/oby.70156 (PMC13250737; doi:10.1002/oby.70156)
Supplement: Supplementary file 1 — Table S1: Demographic Comparison of Participants Completing 18 and 24 months of WW Clinic Participation versus Participants who Discontinued Antiobesity Medication (AOM). Table S2: Monthly Reported Adverse Effects and Prevalence in 18‐Month Cohort. Table S3: Monthly Reported Adverse Effects and Prevalence in 24‐Month Cohort. [file OBY-34-134-s001.docx]

**Supplementary Table S1.** Demographic Comparison of Participants Completing 18 and 24 months of WW Clinic Participation vs Participants who Discontinued Anti-Obesity Medication (AOM)

|  | **Those on AOM at least 18 months** | **Those who discontinued AOM before 18 months** |  | **Those on AOM at least 24 months** | **Those who discontinued AOM 18-24 months** |  |
| --- | --- | --- | --- | --- | --- | --- |
| **Total No.** | n=11,675 | n=1,762 | P-value | n=4,317 | n=529 | P-value |
| **Characteristic** | Mean ± SD; % |  |  |  |  |  |
| **Age, yrs old (SD)** | 43.16 (9.51) | 41.81 (9.83) | <.001 | 43.44 (9.11) | 42.33 (9.72) | 0.010 |
|  |  |  |  |  |  |  |
| **Sex at Birth, %** |  |  | 0.095 |  |  | 0.175 |
| Female | 87.91% | 89.43% |  | 90.55% | 88.75% |  |
| Male | 12.09% | 10.57% |  | 9.45% | 11.25% |  |
|  |  |  |  |  |  |  |
| **Weight, kg (SD)** | 101.19 (20.13) | 102.38 (21.65) | 0.104 | 102.21 (20.81) | 102.38 (20.74) | 0.830 |
| **BMI (SD)** | 36.47 (6.29) | 36.76 (6.46) | 0.146 | 37.01 (6.55) | 36.69 (6.39) | 0.302 |
|  |  |  |  |  |  |  |
| **No. of previous diets tried, (SD)** | 2.91 (1.18) | 3.00 (1.19) | 0.02 | 2.95 (1.18) | 2.88 (1.21) | 0.167 |
| **Type of weight loss tried in the past, %** |  |  |  |  |  |  |
| Dieting | 99.12% | 98.69% | 0.264 | 99.14% | 98.57% | 0.167 |
| Exercise plan | 91.63% | 91.21% | 0.751 | 91.98% | 91.96% | 0.995 |
| Coaching | 53.46% | 54.75% | 0.355 | 55.19% | 55.18% | 0.978 |
| Medications | 39.96% | 41.81% | 0.204 | 44.30% | 40.00% | 0.048 |
| Bariatric surgery | 7.43% | 8.91% | 0.089 | 7.46% | 7.32% | 0.902 |
| Other | 8.90% | 10.10% | 0.148 | 9.22% | 10.54% | 0.314 |
|  |  |  |  |  |  |  |
| **Previous Diagnosis, %** |  |  |  |  |  |  |
| Type 2 Diabetes | 3.19% | 2.85% | 0.754 | 3.76% | 5.19% | 0.124 |
| Obstructive sleep apnea | 10.59% | 12.53% | 0.130 | 11.03% | 10.38% | 0.641 |
| Low HDL | 5.48% | 5.46% | 0.936 | 5.31% | 5.38% | 0.915 |
| High triglycerides | 16.07% | 16.25% | 0.766 | 15.44% | 12.12% | 0.047 |
| Prediabetes | 16.78% | 16.87% | 0.996 | 16.16% | 15.96% | 0.910 |
| Hypertension | 23.18% | 25.68% | 0.112 | 22.35% | 22.69% | 0.835 |
| Heart disease | 0.86% | 0.62% | 0.449 | 0.88% | 0.38% | 0.238 |
| Polycystic ovarian syndrome ^a^ | 12.22% | 16.30% | 0.001 | 13.77% | 13.03% | 0.919 |
| Osteoarthritis | 3.57% | 4.34% | 0.239 | 3.30% | 3.85% | 0.537 |
| Non-alcoholic fatty liver disease | 4.09% | 4.09% | 0.926 | 4.31% | 3.85% | 0.603 |
| Urinary incontinence | 1.26% | 1.25% | 0.954 | 2.32% | 1.15% | 0.084 |
| Gastroesophageal reflux disease | 5.74% | 4.09% | 0.078 | 10.34% | 5.96% | 0.001 |
| None of these | 44.30% | 40.94% | 0.062 | 42.85% | 45.19% | 0.308 |

*a Only females included*

**Supplementary Table S2:** Monthly Reported Adverse Effects and Prevalence in 18-Month Cohort


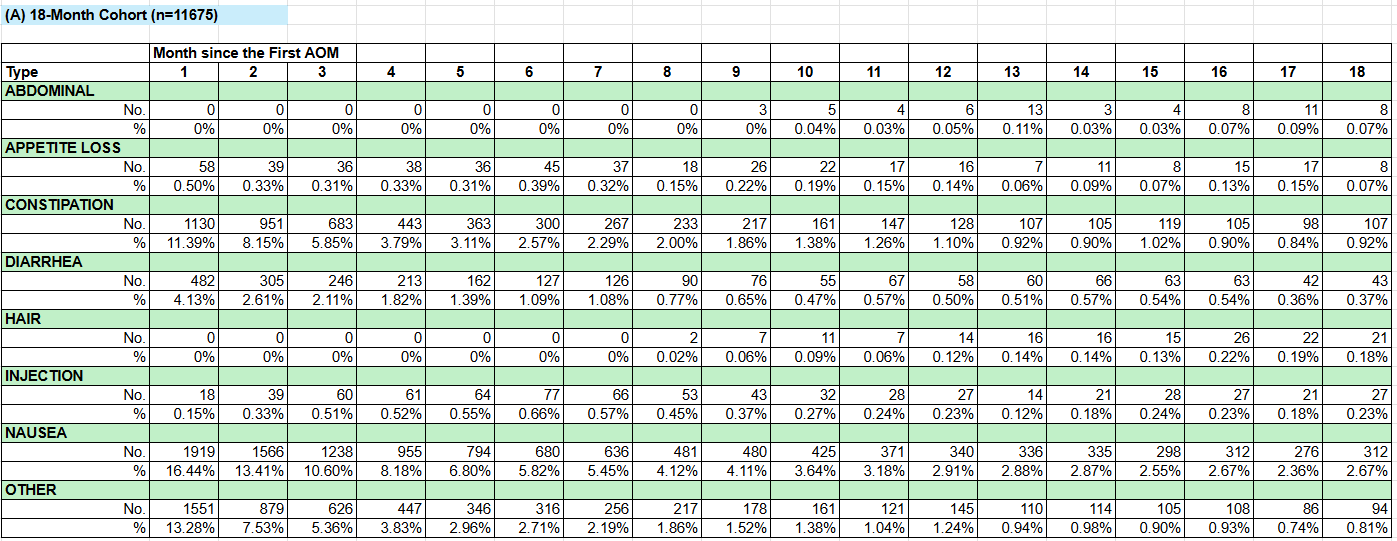


**Other side effects included: heartburn/reflux, fatigue, headache, dizziness, muscle aches, and sleep disturbances*

**Supplementary Table S3:** Monthly Reported Adverse Effects and Prevalence in 24-Month Cohort


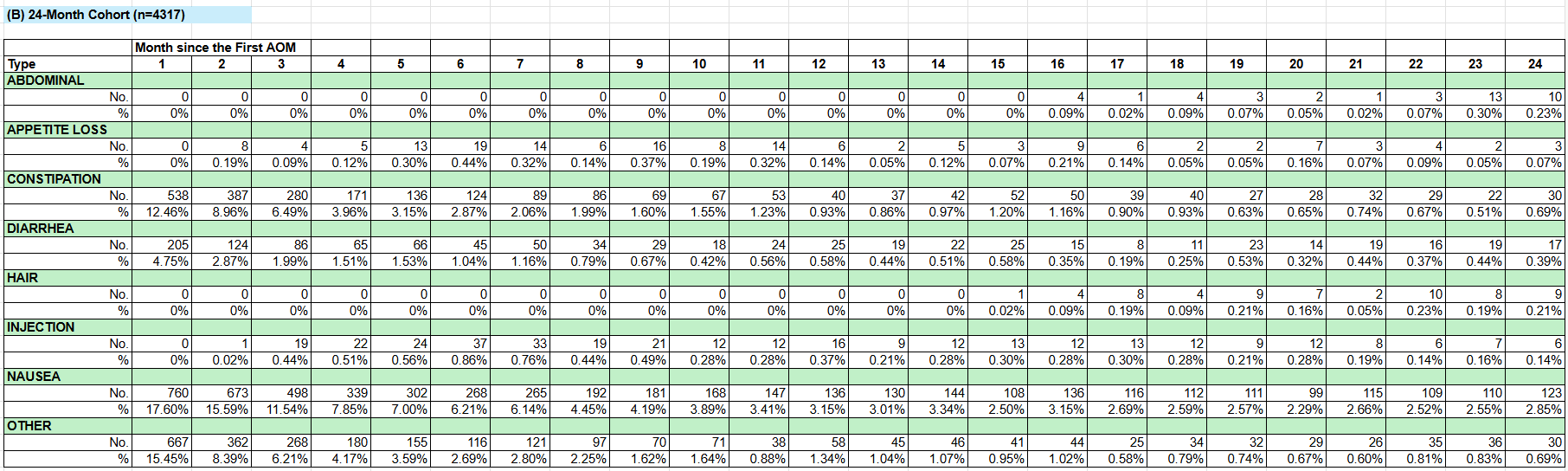


**Other side effects included: heartburn/reflux, fatigue, headache, dizziness, muscle aches, and sleep disturbances*
